# Stable In-Planta Transformation System For Egyptian Sesame (*Sesamum indicum* L.) cv. Sohag 1

**DOI:** 10.1080/21645698.2022.2150041

**Published:** 2023-01-12

**Authors:** Esraa A. A. Sultan, Mohamed S. Tawfik

**Affiliations:** Dept. of Gene transfer, Oil Crops Biotechnology Lab, Agricultural Genetic Engineering Research Institute (AGERI), Agriculture Research Center (ARC), Giza 12619, Egypt

**Keywords:** Agrobacterium, basta, direct transformation, sesame, sesamum indicum

## Abstract

Sesame (*Sesamum indicum* L.) is an important oil crop and one of the oldest-known oil crops to humankind. Sesame has excellent nutritional and therapeutic properties; it is rich in important fatty acids, protein, fiber, and vital minerals. Oil percentage varies among different genotypes but generally accounts for more than 50% of the seed’s dry weight. To meet the increasing demand for vegetable oil production worldwide, expanding the cultivation of oil crops in newly reclaimed areas worldwide is essential. Molecular breeding is an expeditious approach for varietal improvement but requires efficient transgenesis. Published sesame transformation methods are highly genus-specific, tedious, and involve preparing and testing different media and explants. We produced transgenic sesame plants using a stable, noninvasive, and robust *Agrobacterium tumefaciens* transformation method. Leaves and flowers excised from the T_0_ plants at different developmental stages were PCR screened, and 61/93 seedlings were found to be PCR positive. T_1_ seeds resulting from two lines were germinated in a biocontainment greenhouse facility and screened using PCR, basta leaf painting, and spraying fully matured plants with basta herbicide (0.02 mg/l); non-transgenic segregants and control non-transgenic plants were severely damaged, and eventually died, while transgenic plants were not affected by the Basta spraying. RT-PCR on T1 plants indicated the presence of Bar transcripts in T_1_ progeny. Furthermore, RT-PCR using NPTII primers did not result in any amplification in transgenic sesame plants (NPTII is present in the vector but not in the T-DNA region) indicating that the transgenic sesame plants were not an *Agrobacterium*-contaminant.

## Introduction

Sesame is an essential oil crop and was first cultivated in Africa, then spread to India^1^. The Pedaliaceae family contains 17 genera and about 60 species; the *Sesamum* genus has approximately 38 species.^2^ Sesame is rich in oil, which consists of unsaturated fatty acids (83–90%), mainly linoleic acid (37–47%), oleic acid (35–43%), palmitic (9–11%), and stearic acid (5–10%) with a trace of linolenic acid.^3,4^ The cultivation of sesame worldwide is threatened by the susceptibility of commercially available genotypes to biotic, abiotic, and physiological issues that negatively affect plants from reaching their yield potential. Furthermore, breeding efforts in sesame are lagging due to postfertilization problems associated with introducing wild-sesame genotypes into breeding programs.^1^ Analysis of expansion in the cultivated area of oil crops worldwide^5^ indicates that over the past 20-year span (from 2000 up to 2020), expansion in sesame cultivation was less than 2.5 million hectares, compared to an increase of 8.6, 12, and 49.25 million hectares in sunflower, canola, and soybean, respectively (all of which with a well-established track in regeneration/transformation protocol).

Success in transgenesis in plants has been the driving force behind improvement of different crops *via* modern biotechnology worldwide. However, the major obstacle in successfully delivering genes into a plant is the amenability of the target species to regeneration and transformation.^6–8^ Four decades of modern biotechnology have shown that not all economically important crops are regeneration/transformation-friendly; some are more difficult or recalcitrant than others. Establishing a regeneration and transformation system in any given plant is a prerequisite to speed up genetic modification and has been an essential point behind molecular improvements of crops worldwide. Yet, the disadvantages of tissue culture-based transformation protocols are that they are highly genotyped dependent, time-consuming, and the somaclonal variation of putative transgenics affects plants’ qualitative and quantitative characteristics.^9,10^

A valuable alternative to tissue culture-based transformation techniques, especially in species that are difficult to regenerate or immune to transformation, is the In-planta direct transformation. *In-planta* transformation tends to be direct, produces uniform plants, and suits less equipped labs. One of the advantages of using *Arabidopsis thaliana* as a model plant for reverse genetics studies is its *in-planta* transformation relative easiness “*via* floral-dipping technique with vacuum infiltration.”^11,12^ Therefore, it is no surprise that different workers over the past two decades turned their attention to establishing *In Planta* transformation techniques (summarized in Table 1) to overcome tissue culture associated problems.

The establishment of a sesame regeneration system has been problematic, and therefore, sesame was labeled a recalcitrant plant,^13,14^ with a minimal-successful rate of genetic modification.^14,16–18,19,20^ In the present work, we report a new, more straightforward, non-tissue culture-based method for rapid and stable transformation of sesame seeds using the ‘imbibed seed co-cultivation method’ (ISCM). Putative transgenic individual sesame plants were produced and were verified using PCR in T_0_ plants. T_1_ progeny of the two lines were screened using PCR, RT-PCR, leaf painting, and plant spraying with Basta® in biocontainment greenhouse facility. Furthermore, preliminary transcriptome analysis on false septa tissues excised from sesame capsules of transgenic and non-transgenic plants revealed a 100-fold decrease in absolute transcription values of the polygalacturonase gene false septa tissues derived from transgenic plants when compared to non-transgenic control plants.

## Material and Methods

**Materials: *Sesame seeds***: Sesame seeds cultivar Sohag 1 were obtained from the oil crops research division, Crops Research Institute (CRI), Agricultural Research Center (ARC), Giza, Egypt.

**Figure 1.**
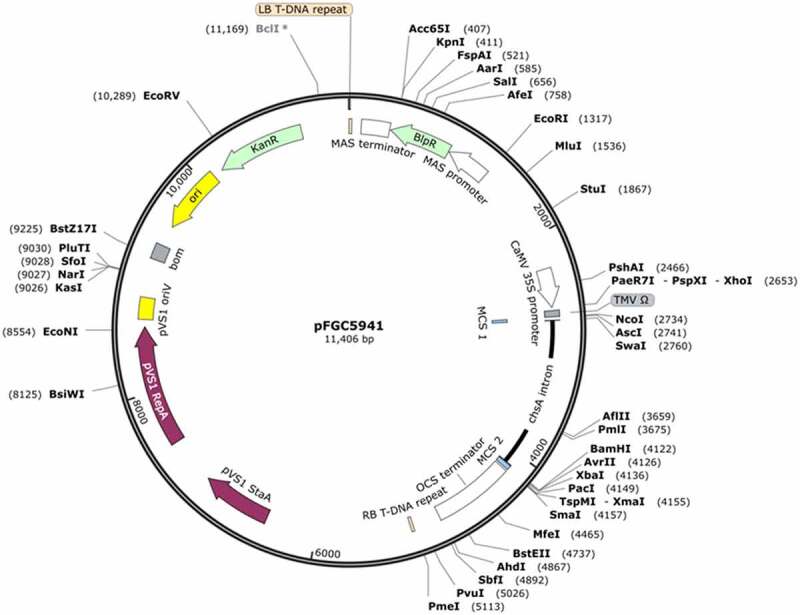
Diagram representing the pFGC5941 plant expression vector that was used to transform sesame plants. The vector contain the bar selectable marker LB: left border repeat from nopaline C58 T-DNA; Kanamycin resistance (KanR) gene for bacterial selection, a basta resistance (BAR) gene for plant selection, a CaMV 35S promoter followed by multiple cloning sites, and a 1,352 bp ChsA intron (from the petunia Chalcone Synthase A gene) to stabilize the inverted repeat of the target gene fragment. RB: right border repeat from nopaline C58. T-DNA (the map was created by SnapGene).

**Methods: *Seed sterilization***: Sesame seeds were surface sterilized in 70% (v/v) ethanol for 1 min, then soaked in 25% commercial bleach with 2–3 drops of Tween®20 followed by placing in a shaker incubator for 15 min. The seeds were rinsed 4–5 times using sterile distilled H_2_O. The seeds were then incubated in ddH_2_O for 10 mins before removing the seedcoat using a sharp scalp. ***Agrobacterium strain and vector***: The pFGC5941 construct (Fig. 1) containing the *bar* gene was transformed into *Agrobacterium* strain LBA4404 *via* heat-shock technique,^21^ and ^22^ and the transformed *Agrobacterium* strain was used for sesame transformation. ***Preparation of Agrobacterium***: A single colony of *A. tumefaciens* strain LBA4404, harboring pFGC5941 construct, was cultured in 25 ml of LB broth medium (containing 50 mg/ml kanamycin, 100 mg/ml streptomycin, and 25 mg/ml rifampicin). The culture was overnight incubated in a shaker incubator at 28°C. The next day, the bacterial culture was centrifuged at 7000 rpm for 5 mins, and the *Agrobacterium* pellet was resuspended in 20–30 ml sterilized water to an optical density (O.D.) of 0.5. ***Sesame transformation procedure***: De-coated sesame seeds were placed on a sterilized plate. Minor scratches were introduced into the embryos using a sharp scalp. The seeds were then placed in a Falcon tube, and 20–25 ml of ready-to-go *Agrobacterium* solution was added. The tube was placed in a shaker incubator at 28°C for 2–3 hr. Followed co-cultivation, seeds were blotted on sterile filter paper and immediately placed on germination medium consisting of ½ strength MS basal salt mixture,^23,24,25^ supplemented with Gamborg’s B₅ vitamin + 10 g/l sucrose + 7 g/l Agar. The cultures were dark incubated overnight at room temperatures. The next day, sesame seeds were transferred into a germination medium supplemented with 500 mg/l cefotaxime. After 5–7 days, the seeds that did not show *Agrobacterium* growth contamination were transplanted into 48-well plastic trays filled with soil (peat moss: clay: sand at 2:1:1 ratio) in growth chambers at 25–28°C with 16/8 hr light/ dark period. Seedlings were kept under these conditions for 2–3 weeks, and then each seedling was transplanted into 15 cm pots filled with the same soil mixture and transferred into a biocontainment greenhouse facility. The seedlings were irrigated and fertilized every 2–3 days with 1.0 g/l MS solution during the first three weeks. ***DNA isolation***: DNA was always isolated from leaves at the apical meristematic part of plants 6, 12- and 18-weeks post transplanting into pots. Flower tissues were also used for DNA isolation in later stages. The leaves were cut into two parts, the 1^st^ part was used for DNA isolation using CTAB [Cetyl trimethyl Ammonium Bromide) method; while the 2^nd^ part was surface sterilized with 70% ethanol for 30 sec, followed by submersing in 10% Clorox solution for 10 mins. The leaves were washed several times with sterilized H_2_O before placing the tissues in plates filled with solid MS medium supplemented with 30 g/l sucrose. The plates were incubated at 28°C for 4 days. Samples that did not show any *Agrobacterium* contamination were used for DNA isolation. DNA was isolated following the procedure of. A 600 µl of CTAB buffer/sample {2% CTAB, 100 mM Tris-HCL (pH 8.0], 1.4 M NaCl, 20 mM EDTA} was preheated in a water bath for 10 mins at 65°C; about 100 mg of sesame leaves were grounded using liquid nitrogen to a fine powder and immediately submerged into the preheated CTAB buffer; 5.0 µl of 0.3% β-mercaptoethanol was added to each tube (containing 600 µl solution) before incubation for 30 minutes at 65°C. Samples were centrifuged at 14,000 rpm for 10 min; then, the upper aqueous phase was transferred to a fresh tube containing 3.0 µl of RNase (Thermo scientific, USA, Cat No. R1253) and incubated at 37°C for 30 min. Chloroform: Isoamyl alcohol (24:1) was added to each tube, mixed well, and then centrifuged at 14,000 rpm for 15 min. The upper phase was transferred to a fresh tube containing 600 µl of ice-cold isopropanol. The tubes were gently mixed, followed by centrifugation at 14,000 rpm for 15 min. DNA pellet was collected and was washed twice with 1.0 ml 70% ethanol followed by centrifugation at 14,000 rpm for 10 min. The ethanol was discarded, and the pellet was allowed to dry completely before 30 µl nuclease-free H2O was added. DNA concentration and quality were determined and measured using nanodrop (BioRad, USA, Cat no. AM 1907, at 260 nm and 280 nm) and were visualized using 1.0% agarose gel. ***Seed setting***: The plants were allowed to grow in big pots in biocontainment facility greenhouse, fertilization was carried out twice a week using 1.5 g/l Kristalon™ (18:18:18 + 3MgO + Micronutrients), additional phosphate fertilization (2.0 g/pot) was added to soil surface once every ten days when flower buds immersed. PCR-positive plants were transplanted into 20 cm pots filled with soil mixture (same as above) and transferred into specially designed mesh-screen boxes in an open field for flowering and seed production. Irrigation was conducted every 3–4 days. Individual PCR-positive plants were transferred into individual cages for seed setting; successful seed production was confirmed in 5 PCR-positive individual T_0_ plants. ***Screening of T_0_ transgenics***: Individual putative transgenic plants were screened using 35S forward and reverse primers (35SF GCTCCTACAAATGCCATCA, and 35SR GATAGTGGGATTGTGCGTCA) along with Bar forward and reverse primers, (Bar F GACAAGCACGGTCAACTTCC, and Bar R CTTCAGCAGGTGGGTGTAGAG), which amplified a 200 and 247bp fragments, respectively. The screening was conducted for T_0_ plants at 6-, 12-, and 18-weeks old plants and from flower tissues with 35S primers. The Bar primers were used from hereon to screen T_1_ seedlings. ***Screening of T_1_ progeny***: Seeds derived from three families (based on seed availability) were tested by planting individual seeds on MS medium. Individual seedlings with strong rooting system were transplanted into 20 cm pots filled with soil mixture. Three weeks post transplanting, 300 mg leaf tissues were collected from each plant and were spelt into two parts (similarly to what has been described previously), the 1^st^ part was used for DNA isolation and PCR screening using Bar forward and reverence primers; the 2^nd^ part was placed on germination medium. The medium was incubated at 28°C for 4 days. ***Basta leaf-painting***: healthy mature, and fully expanded sesame leaves were selected and painted with Basta solution {0.02% Basta and 0.1% (v/v) Tween 20®} using a painting brush. The leaves were observed and scored three days post painting. ***Plant spraying with Basta herbicide***: fully matured sesame T_1_ individual plants, along with non-transgenic segregants AS and non-transgenic controls C, were allowed to grow and reach maturity in biocontainment greenhouse facility. Upon reaching flowing and seed setting stage, a basta solution {0.02% basta with few drops of Tween 20®} was prepared and was used with sesame plants. The plants were sprayed and were screened for yellowish and leaf defoliation symptoms six days post spraying. ***RNA isolation***: RNA from sesame tissues were isolated using PureLink RNA Kit (Ambion, USA, Cat # 12183018A) following the manufacturer protocol. Tissues were grinded in liquid nitrogen and a 100 mg tissue was used for RNA isolation. The RNA was dissolved in 30 µl RNase-Free water. RNA quality and concentrations were determined using nanodrop (Applied Biosystems, at 260 nm and 280 nm) and samples were visualized using 1% agarose gel. ***DNase treatment***: RNA samples were treated with Turbo DNA-free™ (Ambion, USA, Cat # AM1907) to clean samples from any DNA contaminants, following the manufacturer protocol. In a fresh tube the following were added (0.50 µl Turbo enzyme + 5.0 µg RNA sample + 2.5 µl 10 X buffer) total volume was adjusted to 25 µl. The samples were incubated at 37°C for 30 mins, followed by the addition of 2.5 µl DNase inactivation reagent and incubation for 5 mins at room temperature. Samples were centrifuged at 10,000 rpm for 2 mins. The supernatant was then used for cDNA synthesis. ***1^st^ & 2^nd^ strand synthesis***: First strand cDNA synthesis was carried out following the manufacturer’s catalog. In fresh sterile RNase-free microcentrifuge tubes, the following components were added for each sample (5.0 µl RNA + 1.0 µl Oligo dT + 1.5 µl dNTPs + 7.5 µl H_2_O). The tubes were incubated at 70°C for 5 mins, followed by immediate cooling on ice for 60 sec. For each tube 0.4 µl RNase inhibitor + 1.0 µl MMLV enzyme (Promega, USA, Cat no. M170B) + 4.0 µl MMLV 5X buffer were added. Tubes were incubated at 42°C for 5 min, and then an addition 1.0 µl MMLV enzyme was added to each reaction and incubated for 60 mins at 42°C, followed by 5 mins incubation at 70°C. ***The PCR step***: the PCR was carried out using the Bar forward and reverse primers and NPTII primer (NPT II FP CGCAGAAGGCAATGTCATAC and NPT II RP ACCGCTGCGTAAAAGATACG), which amplified 247 bp and 624 bp fragments, respectively. ***Statistical analysis***: Segregation ratio analysis and statistical validation were performed using the chi-square test (Jeliinski et al, 1991).

## Results and Discussion

The need for a non-tissue culture-based transformation protocol in sesame was long felt due to sesame’s recalcitrant nature for *in vitro* regeneration/transformation .^14,15^ All previously successful transformation efforts in sesame came through *in vitro* regeneration,^14,16–18^ with a success rate that varied dramatically along with the different reports (Table 2). In this context, successful *in planta* transformation of sesame could offer a fast, less tedious technique to introduce agronomically important genes to improve essential traits in sesame, as well as dissecting and study the function of genes involved in fatty acid production, oil content, plant–pathogen interaction, and biotic and abiotic stresses.

## Production of Transgenic Sesame T_0_ Plants

**Figure 2.**
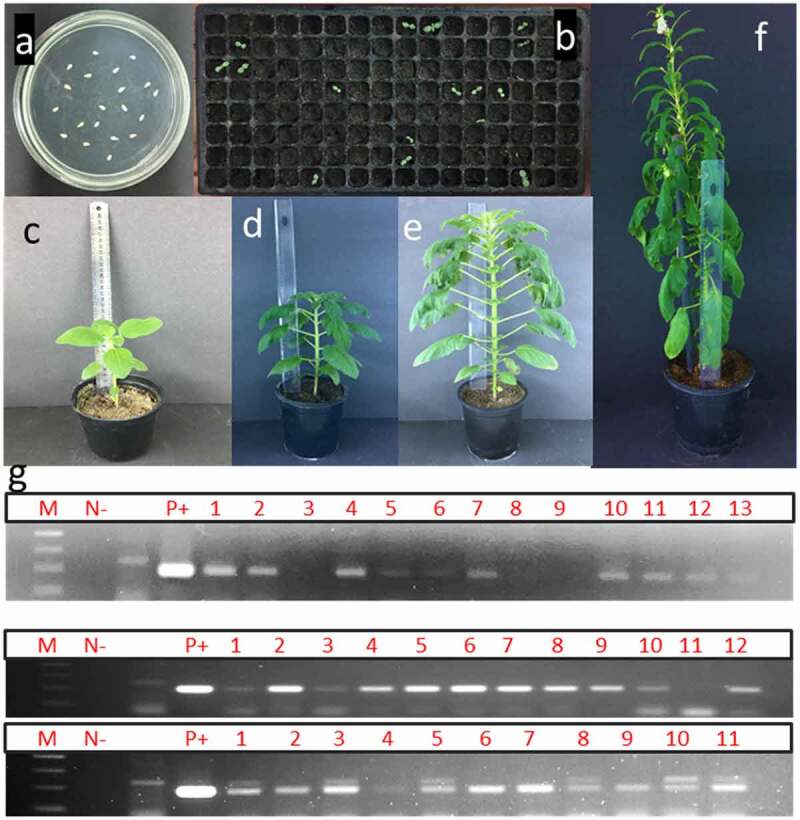
Different stages of transformation of sesame. a) De-coated sesame seeds 48 hr after co-cultivation with Agrobacterium tumefaciens, the seeds are placed on germination medium; b) potential transgenic sesame seeds after being transplanted into trays filled with soil mix in the greenhouse; c) transplanting of successful PCR-positive individuals in bigger pots “plant height 10 cm long”; d) and e) growth of PCR-positive transgenic individual “50 cm and 90 cm, respectively; f) transgenic individual plants reaching flowering and seed setting in greenhouse; g) PCR screening of putative T0 plants from 6, 12, and 18 weeks old plants by 35s primer which amplified 200 bp. M: 100bp ladder DNA marker, lane 1: negative control: water, lane 2: negative control (non-transgenic plant), lane 3: positive control (pFGC5941 RNAi vector), and other lanes transgenic plants.

The entire process of sesame transformation is presented in Fig. 2. Sesame seeds were soaked in water for up to an hour to help seed imbibition and facilitate the seed coat’s removal. Upon removing the seed coat, minor scratches were introduced to the embryonic axes (embedded between the two halves of the cotyledons). The seeds were incubated with *Agrobacterium tumefaciens* strain LBA4404 harboring the pFGC5941 vector (carrying the *bar* gene). *Bar* gene has been used extensively as a selectable marker in previous works (Table 1). The seeds were then transferred onto a germination medium supplemented with cefotaxime for 24 h and then immediately transplanted into soil-filled trays in growth chambers (Fig. 2b).

Transformation experiments were conducted four times, with 1000 seeds (≈ 250 seeds/experiment). In total, 93 seeds developed into fully-healthy matured sesame plantlets (9.3%), of which 61 individuals were found to be PCR-positive (Fig. 2g), an efficiency of more than 67% of the germinated seedlings (an overall efficiency of 6.1%). The first reports on sesame transformation could only report a low percentage of success, ≈ 2%,^16,and14^ with a higher percentage of success in more recent reports.^17,and18^ Interestingly, in both reports, a long, tedious tissue culture-based technique is required with pre-culture treatments, which takes 65–70 days before transplanting seedlings into the soil,^17^ compared to 6–7 days before the emergence of seedlings in a growth chamber in the present work.

Efficient selection of transformed cells and tissues is a critical step in any plant genetic transformation protocol.^26,27^ In most experiments, antibiotic resistance gene(s) such as neomycin phosphotransferase II (*NPT II*) and hygromycin B (*HPT*) have been extensively used. One precise observation when screening previous *in-planta* transformation reports is the choice of the selectable marker (Table 1); most workers opted to use a combination of *NPT II, HPT*, or *Bar* genes as a selectable marker. We opted to use the *Bar* gene in the present work because of its effectiveness as a selectable marker and relative easiness to spray it over plants, and its low cost compared to other selectable agents.

**Figure 3.**
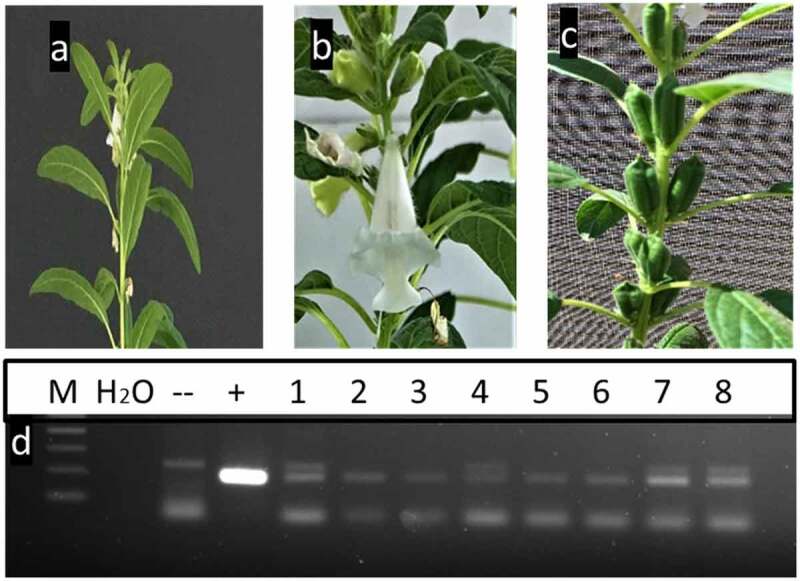
Different developmental stages of putative transgenic sesame plants. a) flowering initiation in sesame plants about 45 days post transformation; b) flowering in putative transgenic sesame plants 65 days post transformation; c) transgenic individual plants reaching seed-setting under greenhouse conditions; and d) PCR-screening of putative transgenics sesame plants using flower-tissues by 35 s primer. M: 100bp ladder DNA marker, lane 1: negative control: water, lane 2: negative control (non-transgenic plant), lane 3: positive control (pFGC5941 RNAi vector), and lane from 4 to 11 transgenic plants.

The putative T_0_ plants that resulted from the in-planta transformation protocol were first subjected to four rounds of PCR screening using *35S* promoter-specific primers. The reason behind the several rounds of PCR screening is to make sure that all the tissues coming from apical meristem are transgenic. Unlike plantlets resulting from *in vitro* regeneration-mediated transformation techniques, the individual sesame plants emerged following the in-planta transformation protocol could produce chimeric tissues.^28^ Therefore, it is essential to have multiple rounds of screening for individual putative transgenics at different developmental stages/time intervals (Fig. 2g). An extra precautionary step was also performed to eliminate possibilities of *Agrobacterium*-contamination in leave tissues, by splitting the tissues to be used for DNA isolation into two parts and placing one part after surface sterilization on germination medium (supplemented with sucrose) followed by incubation at 28C for 4 days.^29,30^ Fresh DNA was isolated from apical leaves of 6, 12- and 18-weeks post transplanting into pots, as well as from flowers (Fig. 3d).

## Segregation Analysis of Transgene in T_1_ Plants

**Figure 4.**
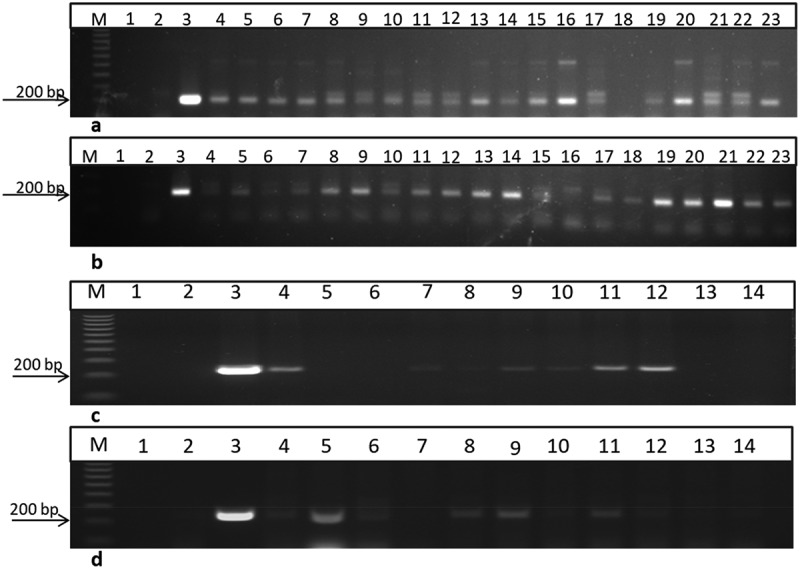
PCR screening of putative transgenic T1 plants from two different lines using 35S primer and bar primers which amplified 200 and 247bp fragments, respectively. a and b using 35S primer lane 1: negative control: H2O, lane 2: negative control (non-transgenic plant), lane 3: positive control (pFGC5941 RNAi vector), and other lanes transgenic plants. c and d using bar primer lane 1: negative control (water), lane 2: negative control (non-transgenic plant), lane 3: positive control (pFGC5941 RNAi vector), and from lane 4 to lane 14 transgenic plants. M: 100bp ladder DNA marker.

Screening of T_1_ transgenic plants using *35S* and *bar* primers that amplified 200 and 247 bp fragments, respectively. Segregation analysis was performed by directly germinating 20 and 11 seeds from two families in trays filled with soil in the biocontainment-greenhouse facility. DNA was isolated from the first fully expanded leaf after the apical meristem from each plant. The leaf was split into two halves, the 1^st^ half was used for DNA isolation, while the 2^nd^ half was used for checking contamination with *Agrobacterium*. The DNA was used to perform PCR (Fig. 4). Analysis of segregation values (using χ^2^ test) revealed the incorporation of the 35S promoter into the T_1_ progeny (Jeliinski, 1991), and statistical analysis revealed that both families were in line with a single gene insertion theory, a 3:1 ratio [P-values were 0.04 and 0.037 for families 1 and 2 at p <.05, respectively, using the 35S primers]. Similarly,^16^used PCR to screen T_1_ progeny and suggested a single gene insertion in T_1_ according to Mendelian fashion [3:1] (χ^2^ = 0.07, 0.95 <P <.80) for uidA gene in sesame plants.

## Herbicide Resistance Test

Herbicide leaf painting assay was conducted to assess the sensitivity of sesame plants toward commercial herbicide basta®. The Leaf-painting technique has been used extensively in screening transgenic plants.^31^ Application of the Basta® herbicide causes a dramatic reduction in glutamine and, therefore, an increase in ammonia levels in leaf tissues, which in turn inhibit photosynthesis and leading to cell-death of non-transgenic plants .^32–34,35,36–38^

**Figure 5.**
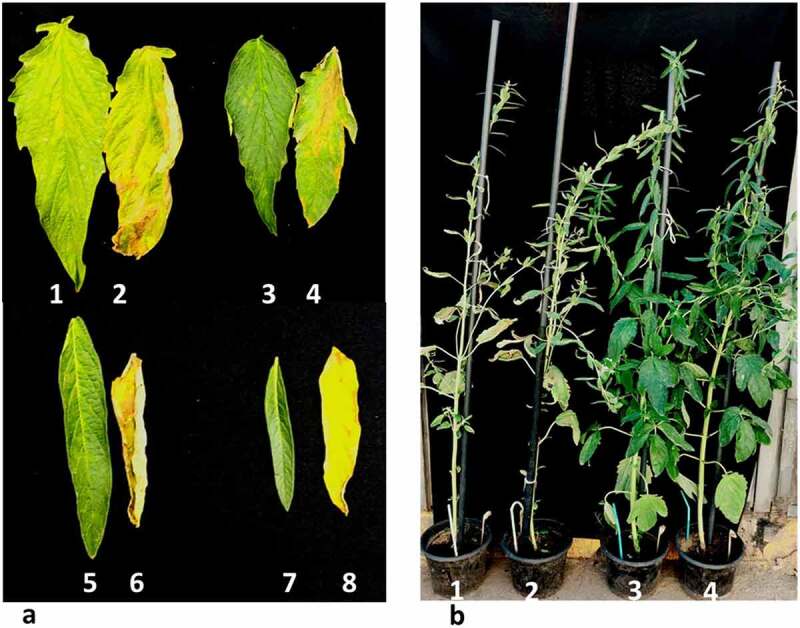
Effect of basta leaf-painting and spraying on sesame plants exhibiting a varying degree of bar gene expression five days post-treatment (0.02% Basta). a. basta-leaf painting derived from transgenic plants (1, 3, 5, and 7) and non-transgenic segregant plants (2, 4, 6, and 8) b. Full-plant spraying with Basta (1) control plant, (2) non-transformed azygous plant, (3) transgenic non-treated plant, and (4) transgenic sprayed plant.

Different Basta® concentrations were tested with sesame control plants (data not shown), we found that a concentration of 0.02 mg/l was sufficient to give intense symptoms within five days post painting (Fig. 5a),^32^selected *Citrullus lanatus* plants by basta leaf painting using a 0.1% (v/v) concentration and reported the death of plants in 3 days. In sorghum plants, a concentration of 0.04% basta solution was used for leaf painting to separate between transgenic and non-transgenic plants used 0.05% basta solution in leaf painting of maize, and non-transgenics individuals showed yellowish of painted parts.

We further tested T_1_, AS, and C sesame plants by spraying fully matured plants with 0.02 mg/l basta® solution (Fig. 5b). The non-transgenic segregant and the control plants exhibited strong yellowing and leaf defoliation symptoms compared to the transgenic T_1_ sesame individuals.

## Screening of Progeny with RT-PCR

**Figure 6.**
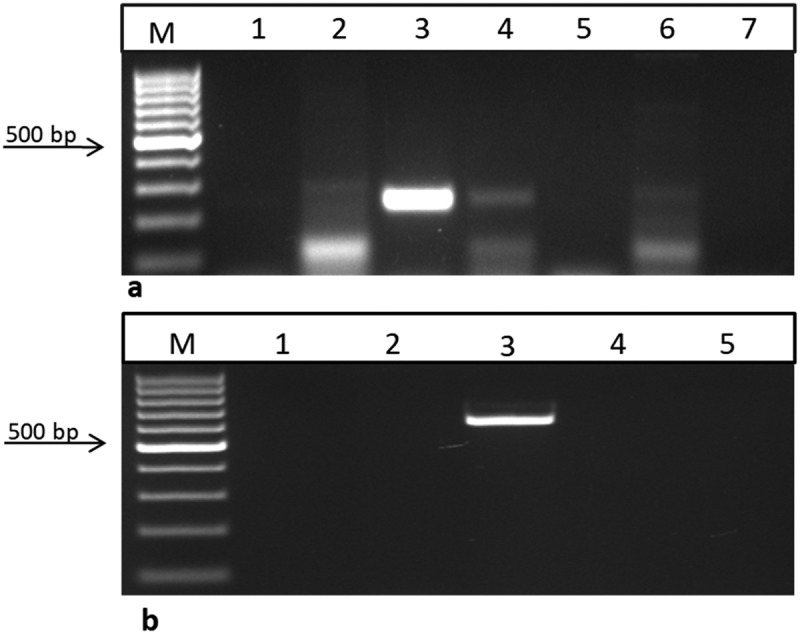
Reverse transcriptase reaction using the bar and nptII primers for RNA samples from T1 sesame plants. a) using bar primer lane 1: negative control (water), lanes 2, 4, and 6: T1 transgenic samples produced a 247bp fragment, lane 3: positive control (pFGC5941 RNAi vector), and lanes 5 and 7: are –RT lines. b) using nptII primer lane 1: negative control (water), lane 2: negative control (non-transgenic plant), lane 3: positive control (pFGC5941 RNAi vector), lanes 4 and 5: RT (transgenic plants from lanes 4 and 6 in the previous gel). M: 100 bp ladder DNA marker, positive sample produced a 680 bp fragment.

^16^carried out RT-PCR analysis to reveal the presence of *uid*A gene in the transgenic sesame plants using *uid*A primer that amplifying 280 bp fragment in all tested T_0_ plants. Similarly,^17^performed RT-PCT for the selectable marker NPTII to confirm the incorporation and expression of the kanamycin selectable marker into sesame, by amplifying a 700 bp NPTII fragment in the four tested individual plants,^18^also performed RT-PCR using bar primers to confirm the incorporation of bar gene in 3 out of the 4 T_0_ plants. Moreover, in the present work, we screened the T_1_ progeny using both NPTII gene (which is present in the *Agrobacterium* vector but not the T-DNA fragment) and Bar gene (present within the T-DNA part). No amplification was observed in all transgenic plants when performing RT-PCR using the NPTII primers, yet a 680 bp fragment was visible in the vector lane (Fig. 6b, lane 3) which clearly indicates that the resulting transgenic sesame plants were not an *Agrobacterium*-contaminant.

The putatively transgenic sesame plants were selected for screening using both BAR and NPTII primers. cDNA libraries were generated from individual plants and were used for RT-PCR. The results confirmed the presence of Bar gene transcripts in positive transgenic T_1_ sesame plants, with the presence of the 247 bp fragment (Fig. 6a). Similarly, previous works by several workers on sesame focused on performing RT-PCR in the presence of the selectable marker transcripts.^16,17,and18^

## Conclusions

We demonstrate the development of transgenic sesame plants for the first time *via* ‘Imbibed Seed Co-cultivation Method,’ an in-planta transformation protocol that has been used over the past few years with some species known to be immune to transformation. Out of a thousand sesame seeds, 93 seeds developed into fully-healthy matured sesame plantlets (9.3%), of which 61 individuals were found to be PCR-positive, a 6.1% transformation efficiency. Transgenic plants from two families were tested, and incorporation of Bar gene into the next generation was verified using PCR, leaf-painting, and spraying of Basta® herbicide of fully matured plants. Non-transgenic control sesame C, and azygos Az sesame plants showed severe yellowish and leaf defoliation upon spraying 0.02% Basta® solution, compared to the transgenic T_1_ sesame plants. Further RT-PCR tests were conducted on T_1_ transgenics and the partial amplification of 247 pb Bar fragment in all transgenic sesame plants was evident. Further analysis using NPTII primers also indicated that no Agrobacterium-contamination was evident in the transgenic individuals. To our knowledge, this is the first report of direct in planta transformation method in sesame using *Agrobacterium*-mediated genetic transformation. This technique, with its low cost, could open the door for advancing the genetic manipulation of sesame to advance human welfare.

## Declarations

1- **Ethics of Conducting Scientific Experiments.**

The manuscript’s author declares that all the experiments and work related to the submitted paper comply with standard ethical procedures for conducting science.

2- **Conflict of Interests.**
